# Comparative Evaluation of a New Depth of Anesthesia Index in ConView® System and the Bispectral Index during Total Intravenous Anesthesia: A Multicenter Clinical Trial

**DOI:** 10.1155/2019/1014825

**Published:** 2019-03-04

**Authors:** Yang Fu, Tao Xu, Keliang Xie, Wei Wei, Ping Gao, Huang Nie, Xiaoming Deng, Guolin Wang, Ming Tian, Min Yan, Hailong Dong, Yun Yue

**Affiliations:** ^1^Department of Anesthesiology, Beijing Chaoyang Hospital affiliated to Capital Medical University, 100020, China; ^2^Department of Anesthesiology, Changhai Hospital Affiliated to Second Military Medical University, 200433, China; ^3^Department of Anesthesiology, Tianjin Medical University General Hospital, 300052, China; ^4^Department of Anesthesiology, Beijing Friendship Hospital affiliated to Capital Medical University, 100050, China; ^5^Department of Anesthesiology, The Second Affiliated Hospital of Zhejiang University Medical College, 310009, China; ^6^Department of Anesthesiology, Xijing Hospital affiliated to The Fourth Military Medical University, 710032, China

## Abstract

The performance of a new monitor for the depth of anesthesia (DOA), the Depth of Anesthesia Index (Ai) based on sample entropy (SampEn), 95% spectral edge frequency (95%SEF), and burst suppression ratio (BSR) was evaluated compared to Bispectral Index (BIS) during total intravenous anesthesia (TIVA). 144 patients in six medical centers were enrolled. General anesthesia was induced with stepwise-increased target-controlled infusion (TCI) of propofol until loss of consciousness (LOC). During surgery propofol was titrated according to BIS. Both Ai and BIS were recorded. Primary outcomes: the limits of agreement between Ai and BIS were -17.68 and 16.49, which were, respectively, -30.0% and 28.0% of the mean value of BIS. Secondary outcomes: prediction probability (Pk) of BIS and Ai was 0.943 and 0.935 (p=0.102) during LOC and 0.928 and 0.918 (p=0.037) during recovery of consciousness (ROC). And the values of BIS and Ai were 68.19 and 66.44 at 50%LOC, and 76.65 and 78.60 at 50%ROC. A decrease or an increase of Ai was significantly greater than that of BIS when consciousness changes (during LOC: -9.13±10.20 versus -5.83±9.63, p<0.001; during ROC: 10.88±11.51 versus 5.32±7.53, p<0.001). The conclusion is that Ai has similar characteristic of BIS as a DOA monitor and revealed the advantage of SampEn for indicating conscious level. This trial is registered at Chinese Clinical Trial Registry with ChiCTR-IOR-16009471.

## 1. Introduction

The accurate and noninvasive assessment of DOA is important for anesthesiologists, and there are several kinds of monitoring devices using electroencephalogram (EEG) signal to provide such information about DOA. EEG reflects cerebral electrical activity over time. During anesthesia, the changes of EEG are nonlinear. Entropy from thermodynamics is then used to explain the DOA, such as response entropy (RE) and state entropy (SE), based on spectral entropy. Because fast Fourier transform (a linear method) is used at the beginning of spectral entropy calculation, some valuable information may be missed [1–3]. Recently, SampEn is used to estimate the complexity and the predictability of EEG signals. The conscious EEG tends to be irregular, which means it cannot be predicted from the previous one and SampEn has a great value. The unconscious EEG tends to be regular, which means it can be predicted from the previous one and SampEn has a small value [4]. The indexes of DOA based on SampEn have better performance than RE, SE, and BIS in predicting consciousness level [3, 4].

It is suggested in previous study that frequency domain analysis of EEG, such as 95%SEF, is suitable to discriminate different anesthesia levels, and time domain analysis, such as BSR, can qualify the extent of deep anesthesia [5]. Based on these three different parameters of EEG: SampEn, 95%SEF, and BSR, a new index of DOA in ConView® system is designed by Pearlcare Medical Technology Company Limited (Zhejiang, China), which is Ai and is calculated with the algorithm based on decision tree and least square [5]. The values of SampEn, 95%SEF, and BSR are treated as inputs, and four different anesthetic levels assessed by experts are treated as outputs. With both of inputs and outputs, the decision tree is trained and modified [5]. In each anesthetic level, the relationship between Ai values estimated by experts and the values of SampEn, 95%SEF, and BRS is almost linear and is fitted with least square. Ai ranges from an isoelectric EEG (0) to a deep hypnotic state (40), general anesthesia (40-60), light/moderate sedation (60-80), and awake (80-99), which is quite the same as BIS does.

BIS is the most widely used DOA-monitoring system and is approved for monitoring hypnosis by the Food and Drug Administration (FDA). It can be a useful monitoring guide for the titration of propofol [6, 7]. And it is the only one that has been studied in large randomized controlled trials, which identified an approximately 80% reduction in the incidence of recall after anesthesia [7]. But it will not predict the exact moment consciousness returns [8]. With the improvement of SampEn in predicting consciousness level, Ai might have better performance than BIS in monitoring DOA.

In this study we tried to evaluate the performance of Ai in predicting anesthetic state compared with BIS during TIVA in six medical centers in China.

## 2. Materials and Methods

This study was approved by the Ethics Committee of Beijing Chaoyang Hospital affiliated to Capital Medical University (No. 2016-ke-100) and registered at Chinese Clinical Trial Registry (No. ChiCTR-IOR-16009471). This comparative evaluation in multicenter was carried out from November 2016 to February 2017. The side of the forehead was randomized on which the EEG electrode strips for Ai or BIS were positioned.

After informed consent, 144 patients (ASA physical state I-II, BMI 18.5-24.9kg/m^2^) aged 18-65 years old and receiving elective surgery under general anesthesia with estimated surgical hours from one to three were enrolled consecutively in each of the six medical centers, which are Tianjin Medical University General Hospital, Beijing Friendship Hospital affiliated to Capital Medical University, Beijing Chaoyang Hospital affiliated to Capital Medical University, the Second Affiliated Hospital of Zhejiang University Medical College, Changhai Hospital Affiliated to Second Military Medical University, and Xijing Hospital affiliated to The Fourth Military Medical University. None of these patients had a medicine history of psychiatric or neurological disorders; impaired cardiac, pulmonary, hepatic, or renal functions; sleep apnea hypopnea syndrome; sedative or analgesic drug therapy or abuse; or contraindication for or allergy to any sedative and analgesic drugs.

EEG electrode strips for recording BIS (BIS XP, system revision 3.31, smoothing rate 15s, Aspect Medical Systems) and Ai (ConView® system, software 2.4.1, Pearlcare Medical Technology Company Limited) were positioned on the forehead cleaned with an alcohol swap, the side of which was randomized by the random numbers from the statistical software. And electrocardiogram, noninvasive blood pressure (NIBP), pulse oximetry, and end-tidal CO_2_ were also monitored. One large vein of the forearm was cannulated with a 18G indwelling needle to administrate drugs.

Oxygen was given by mask. Without premedication, a slow induction was started with 0.01-0.02mg/kg midazolam i.v. push first. Propofol (10mg/ml) was administered i.v. using TCI (Marsh model). Infusion was started at target plasma concentration of 0.5*μ*g/ml, followed by 0.5*μ*g/ml target concentration increase one minute later until LOC [9]. LOC was defined as no response to verbal commands during induction and was tested every thirty seconds. After LOC, remifentanil was applied at 0.2*μ*g/kg/min. Five minutes later, 0.6mg/kg rocuronium was given. And intubation of the trachea was performed one minute later. During surgery, the target plasma concentration of propofol was adjusted to maintain BIS value between 40 and 60, and the infusion rate of remifentanil was titrated to keep NIBP within 1±20% regular NIBP. Rocuronium was added p.r.n. (pro re nata) until thirty minutes before the estimated end of surgery, when 0.1-0.2*μ*g/kg sufentanil was given as the initial postoperation analgesia. After the surgery was finished, propofol and remifentanil infusions were stopped at the same time. ROC was defined as opening eyes following commands and was tested every one minute during emergence.

The values of BIS and Ai were recorded before induction, every one minute while the target concentration of propofol increased until LOC and during the first five minutes of remifentanil infusion, at the time of intubation, and one minute and three minutes after intubation. During the first surgical hour, the values of BIS and Ai were recorded every five minutes and at the time when the infusion rates of propofol or remifentanil were changed based on BIS or NIBP. During emergence, the values of BIS and Ai were recorded every one minute until ROC and one to three minutes after ROC. The target plasma concentration of propofol was recorded at LOC, the end of surgery, and ROC. During data collection, the anesthesiologist estimated the patient's states and recorded the BIS and Ai values at the same time. After data collection, each enrolled patient was assigned a specific number and there was no other patient's identity information involved during data analysis.

Primary outcome was the agreement test of Bland-Altman between Ai and BIS. Secondary outcomes were Pk of BIS and Ai during LOC or ROC and the values of BIS and Ai at 50%LOC, 95%LOC, 5%ROC, and 50%ROC. The sample size in the agreement test of Bland-Altman was suggested to be more than one hundred [10]. It was estimated according to the previous study (n=124) that the performance of Ai was evaluated compared to Narcotrend (not published) and the randomization between left and right sides of forehead used for the EEG electrode strip of Ai among the six medical centers, which should include blocks. The smallest block in this randomization is four. So the sample size in each medical center is 24 and the total sample size is 144.

The agreement test of Bland-Altman is the comparisons of two measurements by bias and precision statistics. The bias is the differences between the two comparative measurements. And with the standard deviation of all the individual bias measurements, the 95% confidence limits are estimated and referred to as the limits of agreement, which is used to judge the precision and acceptability of one measurement against another [10, 11]. The acceptance of a new measurement should rely on limits of agreement being no more than 30% [12]. Pk was used to evaluate how accurately Ai and BIS distinguish conscious and unconscious state [13]. A value of Pk=1.0 means that the index always predicts the conscious state correctly and a value of Pk=0.5 means that the index predicts the conscious state no better than 50/50 chance. Pk and its standard error were calculated with the jack-knife method using a custom spreadsheet PKMACRO in Microsoft Excel 2016 [13]. Pk of LOC was based on all the data during induction and Pk of ROC was calculated using all the data during emergence. Pk was compared with 0.5 using Student's t-test. The difference between these two Pk values of BIS and Ai was studied with paired t-test using another spreadsheet PKDMACRO [13]. And the p values in studying Pk were calculated with the function of TDIST in Microsoft Excel 2016. The relationships between the conscious state and the BIS or Ai values were also defined using logistic regression. Both the BIS and Ai values for 50% or 95% LOC were calculated from the estimated regression equation based on all data during induction. And, based on all data during emergence, so were the BIS and Ai values for 5% or 50% ROC. During LOC and ROC, the changes of Ai or BIS mean values were studied with Wilcoxon test. Data are presented as mean ± SD if not otherwise stated.

## 3. Results

Twenty-four patients for each medical center (144 in total) have accomplished this protocol safely. The males were 41.7% and the females were 58.3%. The age was 44.8 ± 11.8 years and the BMI was 22.8 ± 2.2 kg/m^2^. The left side of the forehead where the EEG electrode strips for Ai were positioned was 52.1% and the right side was 47.9%. In average, the total surgical time was 97.3 ± 35 min. The emergence time was 11.8 ± 7.8 min, which is from the end of anesthetic drugs infusion to ROC. The target plasma concentrations of propofol at LOC, the end of surgery and ROC, and the emergence time for each medical center are shown in Table 1.

**Figure 1:**
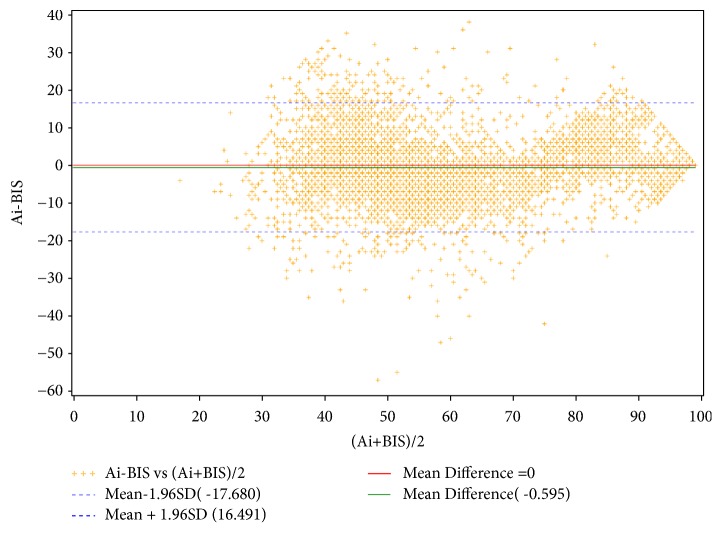
*Bland-Altman plot.* The bias (mean difference) between Ai and BIS was -0.59. The upper limit (mean difference + 1.96SD) was 16.49, and the lower limit (mean difference - 1.96SD) was -17.68. (n=6391).

**Figure 2:**
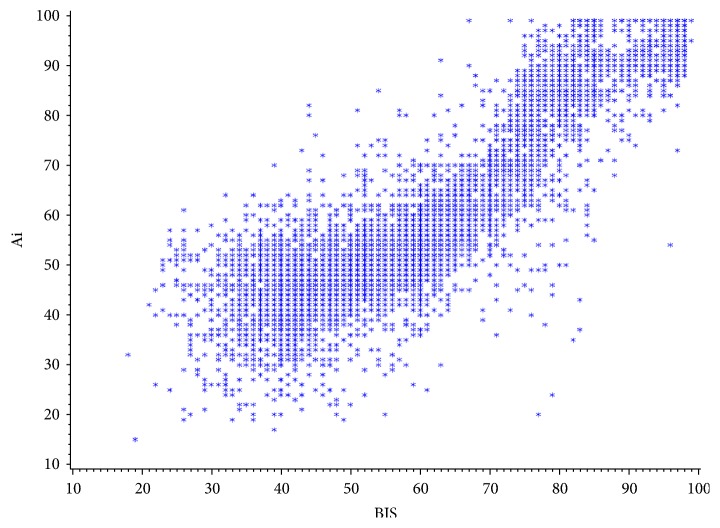
*Ai and BIS plot.* The value of BIS was from 18 to 99. The value of Ai was from 15 to 99. The correlation coefficient between BIS and Ai was 0.873.

The agreement test between Ai and BIS is shown in the Bland-Altman plot in Figure 1. The mean value of BIS was 58.93 ± 17.00 and the mean value of Ai was 58.36 ± 17.50. The bias between Ai and BIS was -0.59 ± 8.72. The limits of agreement were -17.68 and 16.49, which were, respectively, -30.0% and 28.0% of the mean value of BIS. The percentage error (±2SD/mean) was ±29.6%. The relation of BIS and Ai is shown in Figure 2.

Pk values of BIS and Ai are shown in Table 2. All of the Pk values were greater than 0.5. During ROC, Pk of BIS was greater than that of Ai (p=0.037). During LOC, there was no significant difference between the Pk of Ai and BIS (p=0.102).

The BIS and Ai for 50%, 95% LOC and 5%, 50% ROC were calculated from the estimated logistic regression equation and are shown in Table 3.

The values of Ai and BIS during LOC and ROC are shown in Tables 4 and 5. Ai changed far more obviously than BIS from LOC to one minute after LOC (-9.13±10.20 versus -5.83±9.63, p<0.001) and from ROC to one minute after ROC (10.88±11.51 versus 5.32±7.53, p<0.001). The values of Ai and BIS from LOC to three minutes after intubation are shown in Table 6. During the process of deepening anesthesia after LOC, Ai barely changed, which was quite different from BIS.

## 4. Discussions

The variation of the target plasma concentrations of propofol for LOC among medical centers (Table 1) was not noticed until the statistical result revealed it. In this protocol, we tried to define the LOC as concise and practicable as possible. Before starting this study, we checked and discussed every detail of the protocol with the anesthesiologists from different medical centers and performed one together according to this protocol. During carrying out this study, we kept communication with each other in a group by WeChat.

According to the statistical result, the standard deviations among these medical centers are similar, but the mean target plasma concentrations vary a lot. So the differences should be among medical centers and not within each medical center. The lowest concentration is 1.8 *μ*g/ml and the highest one is 3.8 *μ*g/ml. The difference of 2 *μ*g/ml needs four times of concentration increase, which last four minutes, and requires eight times of consciousness checking. Therefore, this big difference comes from not only how we might check LOC differently, but also the different dosages of midazolam (from 0.01mg/kg to 0.02mg/kg), the different kinds of TCI pumps with Marsh Model, and so on. Maybe there is something more important, which we did not find out or we missed.

For the data management, even if there is such obvious difference among medical centers, the data trends of BIS and Ai values are similar during induction, surgery, and emergence. In other words, the quality of anesthesia was maintained well. So the difference of anesthesia among medical centers was finally considered as a new challenge for the agreement test between BIS and Ai, which was not our original intention.

The performance of Ai during the whole surgery with TIVA was evaluated in this multicenter study. The protocol included three components: the slow induction, the first hour of duration of surgery, and the normal emergence. During induction, hypnotics, narcotics, and muscle relaxants were administered one by one and LOC was mainly the result of the accumulating effect of hypnotics. In contrast, ROC during emergence was from the weakening effect of the combination of these components of anesthesia. During surgery the nociceptive stimulations and some kinds of noise such as electrosurgical knife might interfere the EEG monitoring. The differences of anesthesia among the six medical centers (Table 1) might also affect the results of EEG monitoring. All the different situations above were used to evaluate the performance of Ai and to compare the performances of Ai and BIS. So, the agreement test between Ai and BIS included all the data within this protocol. The limits of agreement between the new and the reference technique of up to ±30% are accepted [12], which is the criterion. In this study, the limits of agreement between Ai and BIS are from -30% to 28%, which means that Ai has similar characteristic to BIS index. Pk is a tool to measure the performance of anesthetic depth indicators. In this study, the Pk values of BIS and Ai were 0.943 and 0.935 during LOC and 0.928 and 0.918 during ROC, which means both BIS and Ai were good indicators for consciousness levels. The values of BIS and Ai were 68.19 and 66.44 at 50%LOC, and 76.65 and 78.60 at 50%ROC, which were similar numbers to distinguish consciousness states.

Because it takes time to calculate the properties of EEG signals into BIS values or Ai values, there is some delay when BIS or Ai reveals the information of EEG [14]. Therefore, the change of BIS or Ai from the moment of LOC to one minute after LOC was considered as the response of BIS or Ai for LOC, and so was the change of BIS or Ai during ROC. In this study we found that the change of Ai values during LOC or ROC was greater than that of BIS values (Tables 4 and 5). According to the algorithm of Ai, SampEn is the main component to indicate the change of conscious state [5]. There is a similar finding which suggests that SampEn is more sensitive to the change of conscious state. Shalbaf et al. designed an index of DOA based on SampEn only, which had greater change than SE or RE during LOC and had better performance of estimating the effects of sevoflurane [4]. This might mean that Ai has the advantage of SampEn.

During the slow induction, both the infusion of remifentanil and administration of rocuronium deepened on the anesthesia and prepared the patient for intubation, when Ai barely changed and was quite different from BIS (Table 6). Narcotics in their ordinary doses have no noticeable influence on EEG, but they reduce the change of EEG during nociceptive stimulations [15], which means remifentanil does not cause the difference. The muscle relaxants have no direct action on EEG but they can suppress the activity of frontal electromyogram, which might interfere the EEG measurement [15]. However, after anesthesia induction, Ai has declined markedly and muscle relaxants may no longer have a more pronounced effect.

There are some limitations in this study. During the first surgical hour, the resistance of Ai to the noise which might interfere with the EEG monitoring was meant to be estimated. The duration of interfering was short, the comparison of which needs to be processed in real time. The interval of data points in this study was five minutes and was too long to estimate the resistance of Ai to the noise. Besides, if the interval of data points was shorter during induction or emergence, the change of Ai and BIS at LOC or ROC might be presented in more detail.

Furthermore, as cerebral maturation, the awake resting EEG changes according to the age of child [15], and for a given BIS level, the target concentration of propofol infusion actually decreases as the age increases [16]. So the result of this study focused on the age of 44.8 ± 11.8 could not be extended to children.

Compared with propofol, sevoflurane has quite a different EEG profile. During induction with sevoflurane, the EEG has a biphasic change, which is an increase in fast rhythms followed by a decrease in fast rhythms associated with a simultaneous increase in delta activity [17], revealing an paradoxical increase in BIS during incremental sevoflurane inhalation. So, similarly the result based on propofol infusion could not be extrapolated to inhalational anesthetics either.

## 5. Conclusions

In this study, the performance of Ai was compared with BIS in six medical centers. It is found that Ai has similar characteristic of BIS and revealed the advantage of SampEn for indicating conscious levels.

## Figures and Tables

**Table 1:** The target plasma concentrations of propofol and emergence time for each medical center.

medical center	Concentration of propofol (*μ*g/ml)			Emergence time (min)
	LOC	End of surgery	ROC	
1	1.8 ± 0.4	2.3 ± 0.5	1.1 ± 0.3	8 ± 3

2	3.8 ± 0.5	2.0 ± 0.4	0.7 ± 0.2	10 ± 3

3	3.0 ± 0.6	2.5 ± 0.3	1.1 ± 0.2	10 ± 5

4	3.1 ± 0.5	3.0 ± 0.5	0.8 ± 0.2	23 ± 10

5	2.5 ± 0.7	2.3 ± 0.5	1.1 ± 0.4	12 ± 7

6	2.8 ± 0.7	2.6 ± 0.5	1.4 ± 0.3	7 ± 2

Data are presented as mean ± SD.

Medical Center: 1, Tianjin Medical University General Hospital; 2, Beijing Friendship Hospital affiliated to Capital Medical University; 3, Beijing Chaoyang Hospital affiliated to Capital Medical University; 4, the Second Affiliated Hospital of Zhejiang University Medical College; 5, Changhai Hospital Affiliated to Second Military Medical University; 6, Xijing Hospital affiliated to the Fourth Military Medical University.

LOC: loss of consciousness. ROC: recovery of consciousness. Emergence time: from the end of anesthetic drugs infusion to ROC.

**Table 2:** Pk values of BIS and Ai during LOC or ROC.

	Pk	
	Ai	BIS
LOC	0.935 ± 0.005	0.943 ± 0.005

ROC	0.918 ± 0.007#	0.928 ± 0.006#

Data are presented as mean ± SE.

^#^ Difference between BIS and Ai during ROC (p<0.05).

All of the Pk values were greater than 0.5 (p<0.01).

**Table 3:** The values of BIS and Ai at 50%, 95% LOC and 5%, 50% ROC.

	Ai	BIS
50% LOC	66.44	68.19

95% LOC	48.25	52.31

5% ROC	55.72	63.13

50% ROC	78.6	76.65

LOC: loss of consciousness. ROC: recovery of consciousness.

**Table 4:** The values of Ai and BIS during LOC.

	Ai	BIS	Ai-BIS
1 min before LOC	62.85 ± 12.68	64.01 ± 10.82	-1.49 ± 9.05

LOC	60.76 ± 12.37	62.18 ± 10.69	-1.42 ± 9.19

1 min after LOC	51.63 ± 11.48	56.35 ± 8.98	-4.27 ± 9.25

Data are presented as mean ± SD.

Ai changed far more obviously than BIS from LOC to one minute after LOC (-9.13±10.20 VS -5.83±9.63, p<0.001).

**Table 5:** The values of Ai and BIS during ROC.

	Ai	BIS	Ai-BIS
1 min before ROC	69.65 ± 15.25	72.01 ± 9.76	-2.35 ± 9.21

ROC	73.90 ± 13.67	75.66 ± 7.99	-1.75 ± 10.19

1 min after ROC	84.78 ± 9.33	80.98 ± 5.52	3.81 ± 8.31

Data are presented as mean ± SD.

Ai changed far more obviously than BIS from ROC to one minute after ROC (10.88±11.51 VS 5.32±7.53, p<0.001).

**Table 6:** The values of Ai and BIS from one minute after remifentanil infusion to one minute after intubation.

	Ai	BIS	Ai-BIS
R1	51.63 ± 11.48	56.35 ± 8.98	-4.72 ± 9.25

R5	51.38 ± 7.50	52.95 ± 8.60	-1.57 ± 6.25

T0	50.35 ± 8.26	48.92 ± 9.98	1.37 ± 8.73

T1	49.19 ± 8.21	46.71 ± 10.06	2.49 ± 8.48

Data are presented as mean ± SD.

R1: one minute after remifentanil infusion. R5: five minutes after remifentanil infusion. T0: the time of intubation. T1: one minute after intubation.

During the process of deepening anesthesia from R1 to T1, Ai barely changed, which was quite different from BIS (-2.15±12.25 VS -9.58±11.67, p<0.001).

## Data Availability

The data used to support the findings of this study are included within the article.

## Supplementary Materials

Supplementary Materials S1 File. GRRAS checklist. S2 File. Datasets.
