# Supplementary material for: Comparative Evaluation of a New Depth of Anesthesia Index in ConView® System and the Bispectral Index during Total Intravenous Anesthesia: A Multicenter Clinical Trial
Source: Biomed Res Int. 2019 Mar 4;2019:1014825. doi: 10.1155/2019/1014825 (PMC6425335; doi:10.1155/2019/1014825)
Supplement: Supplementary Materials — S1 File. GRRAS checklist. S2 File. Datasets. [file 1014825.f1.zip › 1014825.f1/S1_File.docx]

GRRAS checklist for reporting of studies of reliability and agreement

Version based on Table I in: Kottner J, Audigé L, Brorson S, Donner A, Gajeweski BJ, Hróbjartsson A, Robersts C, Shoukri M, Streiner DL. Guidelines for reporting reliability and agreement studies (GRRAS) were proposed. J Clin Epidemiol. 2011;64(1):96-106

| **Section** | **Item #** | **Checklist item** | **Reported on page #** |
| --- | --- | --- | --- |
| Title/Abstract | 1 | Identify in title or abstract that interrater/intrarater reliability or agreement was investigated. | 1,3 |
| Introduction | 2 | Name and describe the diagnostic or measurement device of interest explicitly. | 4-6 |
|  | 3 | Specify the subject population of interest. | 7-8 |
|  | 4 | Specify the rater population of interest (if applicable). |  |
|  | 5 | Describe what is already known about reliability and agreement and provide a rationale for the study (if applicable). |  |
| Methods | 6 | Explain how the sample size was chosen. State the determined number of raters, subjects/objects, and replicate observations. | 7-11 |
|  | 7 | Describe the sampling method. | 7 |
|  | 8 | Describe the measurement/rating process (e.g. time interval between repeated measurements, availability of clinical information, blinding). | 8-10 |
|  | 9 | State whether measurements/ratings were conducted independently. | 7-8 |
|  | 10 | Describe the statistical analysis. | 11-12 |
| Results | 11 | State the actual number of raters and subjects/objects which were included and the number of replicate observations which were conducted. | 12-15 |
|  | 12 | Describe the sample characteristics of raters and subjects (e.g. training, experience). | 13 |
|  | 13 | Report estimates of reliability and agreement including measures of statistical uncertainty. | 14-16 |
| Discussion | 14 | Discuss the practical relevance of results. | 18-20 |
| Auxiliary material | 15 | Provide detailed results if possible (e.g. online). |  |
